# Prognostic Significance of the Lymphoblastic Leukemia-Derived Sequence 1 (LYL1) GeneExpression in Egyptian Patients with AcuteMyeloid Leukemia

**DOI:** 10.4274/tjh.2012.0063

**Published:** 2014-06-10

**Authors:** Nadia El-Menshawy, Doaa Shahin, Hayam Fathi Ghazi

**Affiliations:** 1 Mansoura University Faculty of Medicine, Department of Clinical Pathology, Mansoura, Egypt; 2 Mansoura University Faculty of Medicine, Department of Oncology Medicine, Mansoura, Egypt

**Keywords:** LYL1 gene, Acute myeloid leukemia, myelodysplastic syndrome, Chronic myelogenous leukemia in blast and accelerated phases, Real-time polymerase chain reaction

## Abstract

**Objective:** Aberrant activation of transcription factor genes is the most frequent target of genetic alteration in lymphoid malignancies. The lymphoblastic leukemia-derived sequence 1 (LYL1) gene, which encodes a basic helix-loop helix, was first identified with human T-cell acute leukemia. Recent studies suggest its involvement in myeloid malignancies. We aimed to study the expression percent of oncogene LYL1 in primary and secondary high-risk myeloid leukemia and the impact on prognostic significance in those patients.

**Materials and Methods:** Using quantitative real-time polymerase chain reaction for detection of LYL1 oncogenes, our study was carried out on 39 myeloid leukemia patients including de novo cases, myelodysplastic syndrome (MDS) with transformation, and chronic myelogenous leukemia (CML) in accelerated and blast crisis, in addition to 10 healthy individuals as the reference control.

**Results:** LYL1 expression was increased at least 2 times compared to the controls. The highest expression of this transcription factor was observed in the MDS cases transformed to acute leukemia at 7.3±3.1, p=0.0011. LYL1 expression was found in 68.2%, 75%, and 77.8% of cases of acute myeloid leukemia, CML crisis, and MDS, respectively. Significant correlation of LYL1 overexpression with some subtypes of French-American-British classification was found. There was, for the first time, significant correlation between the blood count at diagnosis and LYL1 expression (p=0.023, 0.002, and 0.031 for white blood cells, hemoglobin, and platelets, respectively). The rate of complete remission was lower with very high levels of LYL1 expression and the risk of relapse increased with higher levels of LYL1 expression, suggesting an unfavorable prognosis for cases with enhanced expression.

**Conclusion:** Overexpression of LYL1 is highly associated with acute myeloid leukemia and shows more expression in MDS with unfavorable prognosis in response to induction chemotherapy. These observations could signal a promising tool for a therapeutic target to basic helix–loop helix protein related to transcription factors, which may improve patient outcome in acute myeloid leukemia, MDS, and CML in blast crisis.

## Abstract (tr)

**Amaç:** Lenfoid malignitelerdeki genetik değişikliğin en sık hedefi transkripsiyon faktör genlerindeki anormal aktivasyondur. Lenfoblastik Lösemi Kökenli Dizi 1 (LYL1) temel bir sarmal-ilmek-sarmal proteini kodlar ve ilk olarak T-hücreli akut lösemili bir insanda tespit edilmiştir. Son yapılan çalışmalar miyeloid malignitelerle olan ilişkisini göstermektedir. Bu çalışmada yüksek riskli birincil ve ikincil akut miyeloid lösemi hastalarında LYL1 onkogeninin ifade edilme yüzdesi ve bunun prognoza olan etkisini göstermeyi amaçladık.

**Gereç ve Yöntemler:** Çalışmamıza; miyelodisplastik sendromdan (MDS) dönüşmüş veya de novo akut miyeloid lösemili veya akselere/blastik evrede kronik miyeloid lösemili (KML) olmak üzere toplam 39 hasta ve kontrol amaçlı 10 sağlıklı kişi dahil edilmiştir. LYL1 gen ifadesi kantitatif gerçek-zamanlı polimeraz zincir reaksiyonu kullanılarak ölçüldü.

**Bulgular:** LYL1 gen ifadesinin kontrollere göre en az 2 kat arttığı görüldü. En yüksek LYL1 gen ifade oranları MDS’den akut lösemiye dönüşen olgularda gözlendi (7,3±3,1; p=0,0011). LYL1 gen ifade oranları akut miyeloid lösemi, KML blastik kriz ve MDS hastalarında sırasıyla %68,2, %75 ve %77,8 bulundu. LYL1 aşırı gen ifadesi ile Fransız-Amerikan-İngiliz (FAB) sınıflaması bazı alt tipleri arasında anlamlı korelasyon bulundu. Literatürde ilk kez, tanı anındaki kan sayımı ve LYL1 gen ifadesi ile lökosit sayısı, hemoglobin düzeyi ve trombosit sayısı arasında anlamlı korelasyon bulunmuştur (sırasıyla, p=0,023, 0,002, ve 0,031). Yüksek LYL1 ifadesinin saptandığı olgularda tam yanıt oranının düşük ve relaps riskinin artmış saptanması, artmış gen ifadesinin olumsuz prognostik özellikte olduğunu düşündürmektedir.

**Sonuç:** LYL1 aşırı ifadesi akut miyeloid lösemi ile yüksek derecede bağlantılıdır. Olumsuz prognostik özellik gösteren MDS hastalarında aşırı LYL1 gen ifadesinin indüksiyon tedavisine ilişkili olduğu görülmektedir. Bu gözlemler transkripsiyon faktörleri ile ilişkili sarmal-ilmek-sarmal yapısındaki proteinlerin birer terapötik hedef olarak gelec vaad ettiğini ve AML, MDS ve blastik krizdeki KML hastalarında prognozu olumlu etkileyebileceğini düşündürmektedir.

## INTRODUCTION

Transcription factors play an important role in the normal developmental process of hematopoietic cells. However, expression of transcription factors and their implications in various human leukemia types are not well understood [1].

Recent research has focused on these oncogenic transcription factors and their role in leukemogenesis. The lymphoblastic leukemia-derived sequence 1 (LYL1) gene encodes a basic helix-loop helix (bHLH) protein with 267 amino acids and a molecular weight of 28.628 Da. It was originally identified in some cases of T-cell acute lymphoblastic leukemia (T-ALL) at the breakpoint region of the chromosomal translocation t (7;19) (q35; p13) [2]. The translocation is in head-to-head juxtaposition with the T-cell antigen receptor beta (TCR-beta) gene, resulting in truncation of the LYL1 gene and production of abnormal-sized RNAs. This brings the LYL1 gene under the regulatory control of the TCR-beta gene, resulting in ectopic expression of LYL1 [3]. However, overexpression of LYL1 has also been reported in cases of T-ALL without apparent chromosome aberration. Interestingly, another study by Kuo et al. showed that LYL1 was expressed in most B-lineage cells but was downregulated during terminal differentiation, whereas most of the T-lineage cells did not express LYL1 [4].

Furthermore, LYL1 mRNA expression has been shown to occur in the developing cardiovascular and hematopoietic system cells, but not in developing nerve cells [5]. LYL1 forms heterodimeric complexes with E2A and p105, the precursor of nuclear factor-kappaB1 p50, and functions as an inhibitor that prevents the activation of E2A-responsive tumor suppressor genes, leading to differential arrest and cell transformation [6].

Some transcription factors, such as TAL1, LYL1, and LMO1/2, are tissue-specific bHLHs that bind to DNA as heterodimers with HEB or one of the E2A proteins and form a multiprotein complex [7]. They are transcribed in immature hematopoietic cells, where they are essential for normal development, but not in the T-lymphoid lineage [8,9].

The bHLH region of LYL1 and TAL1/SCL proteins shows 82% amino acid identity, suggesting that these 2 proteins share at least some target genes and biological functions [10]. 

Chambers et al. found that LYL1 is not essential for embryonic development; however, deletion of LYL1 together with its paralog, the stem-cell leukemia (SCL) gene, causes rapid apoptosis of hematopoietic progenitors in adult mice [11]. The upregulation of LYL1 has been linked to a subtype of T-ALL defined by a stem-like phenotype and an unfavorable prognosis [12]. Excess LYL1 blocked the dimerizations of E2A and thus inhibited the regulatory activity of E2A on the CD4 promoter, leading to increased proliferation and suppressed apoptosis of the progenitor cells [13]. Furthermore, overexpression of LYL1 in mouse bone marrow causes expansion of the hematopoietic progenitors and the mature T-cells. These effects were most likely due to the anti-apoptotic and proliferative roles of the LYL1 overexpression in the hematopoietic system [14].

Ferrando et al. found that expression of LYL1 was increased in immature T cell precursor cells and was associated with unfavorable prognosis in T-ALL cases [15]. Zhong et al. studied the effect of LYL1 in transgenic mice and observed that a significant proportion of the mice developed B and T cell lymphoma after an average latent period of 1 year [16].

However, few studies have looked for the LYL1 expression of leukemic cell lines and clinical cases of myeloid leukemia. The aim of the present study was to investigate the expression rate of the oncogene LYL1 in primary and high-risk myeloid leukemia and to assess its impact on prognosis. 

## MATERIALS AND METHODS

**Patients **

Thirty-nine patients with primary or secondary acute myeloid leukemia (AML) being followed at the medical oncology center of Mansoura University and 10 healthy individuals were included in this case-control study. Twenty-two patients were diagnosed with de novo AML (Group I), 8 patients were in the accelerated and myeloid blast phases of chronic myeloid leukemia (CML) (Group II), and 9 patients had AML transformed from myelodysplastic syndrome (MDS) (Group III). The control group consisted of 10 healthy individuals with normal peripheral blood counts and morphology. Peripheral blood and bone marrow (BM) samples were obtained from healthy controls and patients in accordance with the protocols of the local institutional ethics committee. 

All patients were subjected to complete clinical examination. Diagnosis was based on complete blood count, BM examination, and immunohistochemical, morphological, and immunophenotyping studies of the marrow samples to confirm the specific clonality and correlation with morphological French-American-British (FAB) classification. Patient characteristics are given in Table 1.

**Analysis of LYL1 Expression by Real-Time Polymerase Chain Reaction **

**Sample Collection**

BM aspirates and venous blood samples were collected from each patient with completely aseptic techniques and under mild conscious sedation. Ten peripheral venous blood samples were obtained from the healthy controls to determine the reference level of LYL1.

**Analysis**

RNA was extracted from the blood or marrow cells using a commercial RNA isolation kit (QIAamp RNA Blood Mini Kit, QIAGEN, Hilden, Germany; Cat. No. 52304) according to the manufacturer instructions. The specificity of the LYL1 amplification was confirmed by the observation of a unimolecular dissociation curve and a single band on 3% agarose gel following electrophoresis. 

RNA was reverse transcribed from 1 µg of total RNA in a final volume of 20 µL containing reverse transcription-polymerase chain reaction (RT-PCR) buffer (1 mM each dNTP, 3 mM MgCl2, 75 mM KCl, 50 mM Tris-HCL, pH 8.3), 10 U RNA (Promega, Madison, WI, USA), 100 mM dithiothreitol, 100 U superscript II (Gibco-BRL, Cergy-Pontoise, France), and 25 µM random hexamers. The expression levels of various target PCRs were quantified relative to the expression level of the endogenous housekeeping gene, glyceraldehyde-3-phosphate dehydrogenase (GAPDH), by real time RT-PCR in a Step-One 7000 (PE Applied Biosystems, Foster City, CA, USA) as described previously [9].

For detection of LYL1 expression levels, the forward primer 5`- TCA CCC CTT CCT CAA CAG TGT- 3` and reverse primer 5`- CGG GCC ACC TTC TGG G- 3` were used in combination with the probe 5`- (FAM)- CCT TCA CAC GCC TGC AGA TC- (TAMRA) -3`. Each sample had internal endogenous controls of GAPDH forward primer 5`- GAA GGT GAA GGT CGG AGT C -3` and GAPDH reverse primer 5`- GAA GAT GGT GAT GGG ATT TC -3`.

The cDNA was amplified for 40 cycles of 15 s at 95 °C (denaturation) and 1 min at 60 °C (annealing and extension). The relative copy numbers of gene expression were quantified using the comparative threshold cycle (ct) method as shown in Figure 2. 

**Statistical**

The data collected were statistically analyzed using SPSS 16 (SPSS Inc., Chicago, IL, USA). The Student t-test, the Mann-Whitney U test, and one-way ANOVA (t-test) were used when making comparisons between groups. Correlations among variables were found by using Spearman’s correlation coefficient and linear regression analysis. A 2-sided p value of less than 0.05 was considered significant. Qualitative data are presented as numbers and percentages. Quantitative data are presented as mean ± standard deviation or median (minimum-maximum) where appropriate. 

## RESULTS

Expression of LYL1 was studied in 22 de novo and 17 high-risk AML patients. LYL1 expression levels of the 4 study groups are presented in Table 1.

**Figure 1:**
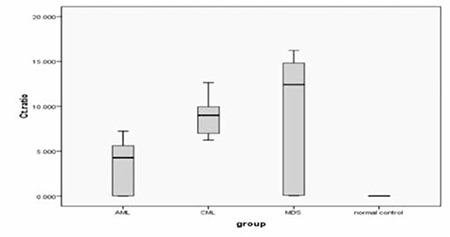
Interaction box plot representing expression level of LYL1 in different patient groups (p=0.000).

LYL1 expression was significantly higher in comparison to controls in all 3 groups with mean values of 4.6±2.9, 6.8±2.1, and 7.3±3.1 for Groups I, II, and III, respectively (related p values were 0.008, 0.003, and 0.0011, respectively). The highest expression level was detected in MDS transformed leukemia patients (Figure 1). 

**Figure 2:**
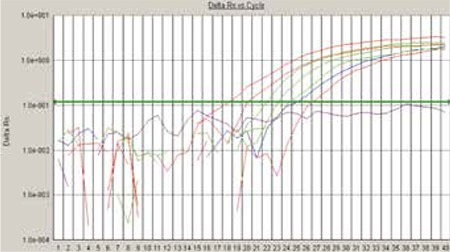
Representing positive LYL1 and positive GAPDH internal control

An increase in expression of at least 2 times the normal control was considered positive for LYL1 expression. Accordingly, positive LYL1 expression was found in 15/22 (68.2%), 6/8 (75%), and 7/9 (77.8%) cases in Groups I, II, and III, respectively (Table 2, Figure 2)

No significant correlation between the expression of LYL1 and the hemoglobin concentration, leukocyte count, platelet count, or number of blasts in the peripheral blood and in the BM could be demonstrated, as shown in Table 3. 

 We also examined whether there was an increased tendency for clustering in a certain FAB subtype in patients with enhanced LYL1 expression (Table 4). In this regard, we observed that patients with increased levels of expression tended to be of the M2, M3, and M6 subtypes more frequently; however, the numbers were too small to present this as a significant relationship. 

Furthermore, we tested whether BM LYL1 expression at diagnosis could predict the response to induction chemotherapy or the achievement of complete remission. We found that 71.4% of patients negative for LYL1 expression achieved complete remission, while 60% of those positive for LYL1 expression reached complete remission (p=0.065). The mean LYL1 expression was higher in the relapsed patients, at 5.7±3.2 (p=0.032). The relapse rate was almost one-third higher in patients with increased LYL1 expression (Table 5).

Correlations of LYL1 expression with clinical and laboratory parameters were studied. LYL1 was not found to be correlated with splenomegaly, lymphadenopathy, or level of peripheral blood or BM blasts at presentation. There was a significant positive correlation between the LYL1 expression and the leukocyte number as well as the age at diagnosis. LYL1 expression was shown to be significantly negatively correlated with the levels of hemoglobin and platelets on admission and the rate of complete remission achievement (Table 6).

## DISCUSSION

The LYL1 gene codes for a bHLH transcription factor [16]. The basic region facilitates DNA interactions, while the helix-loop helix domain enhances protein dimerizations and prevents activation of E2A-responsive tumor suppressor genes, leading to differential arrest and cell proliferation [17]. LYL1 has an important role in hematopoietic stem cell biology and normal hematopoiesis, while its expression has been associated with the development of leukemia [18,19]. 

Many researchers have focused on the expression of LYL1 levels in acute lymphoblastic leukemia, while only a few studies on expression of this factor in AML are available. Our present study aimed to assess the expression of LYL1 in myeloid malignancy, either de novo AML or secondary to MDS or CML. We found increased levels of expression of this transcription factor by at least 2 times that of the controls in the AML group at 68.2%, the MDS group at 77.8%, and the CML group at 75%, while it was expressed at very low levels in the normal control group. Similar results were reported by Meng et al. [20], who observed enhanced expression of LYL1 in 79.2% (19/24) of AML cases and 81.8% of MDS cases (9/11). In another study [21], Meng et al. recently reported a slightly lower overexpression rate of this transcription factor, at 62.2%. This variation of expression may be related to the methodology of different techniques used in the studies or to characteristic features of the study populations. Geographical or racial factors that differ from one population to another may also underlie the different expression frequencies. Furthermore, the expression of LYL1 might be associated with coexpression of other transcription factors, making it difficult to assess. 

The mechanism by which LYL1 is highly expressed in AML is not known, while in T-ALL, it has been explained by its upregulation related to a translocation. However, recent studies revealed that it could also occur without the presence of particular translocations. There are multiple mechanisms responsible for LYL1 upregulation in AML. Chan et al. [22] reported that elongation transcriptions and GATA factors regulate LYL1 transcription. Post-translational processing and degradation are the main regulatory mechanisms controlling protein expression and function. San-Marina et al. [23] concluded that the cAMP regulatory element-binding protein (CREB1), a widely expressed transcription factor and a suspected oncogene in AML, was a binding partner for LYL1. The interaction between LYL1 and CREB1 occurs at the N-terminal domain of LYL1 and the Q2 and KID domains of CREB1. The histone acetyltransferase p300 and the core-binding protein are recruited to these complexes, leading to Ser 133 phosphorylation and direct transcriptional activation. The ability of LYL1 to modulate promoter responses to CREB1 suggests that it might play a role in the malignant phenotype. Lukov et al. proposed in their study that post-translational mechanisms for upregulation of LYL1 might contribute to its oncogenic role [24]. 

We observed higher overexpression of LYL1 in MDS compared to de novo AML cases. This suggests that involution chromosomal abnormalities may contribute to oncogenic pathogenesis in MDS patients. These findings confirmed the aberrant expression of LYL1 in AML and MDS, first by increased expression in the stem population and then by persistent expression in the downstream progeny. Meng et al. observed that CD34-enriched AML cells had a slightly higher level of LYL1 expression in the AML population. This indicated that LYL1 was highly expressed in the stem cell fraction, while Lukov et al. [25] found increased proliferation and suppressed apoptosis of progenitor cells and presented substantial evidence supporting the pro-leukemic effect of LYL1 in early hematopoietic progenitors with the potential to cause expansion of malignant cells with a stem cell/early progenitor-like phenotype. Therefore, deregulated transcription and its consequence on key development pathways seems to play a major role in the molecular pathogenesis of lymphoid malignancy, as concluded by O’Neil and Look [26]. However, further research is required to explain this finding in myeloid malignancies, especially MDS. In regard to our observation of increased expression of LYL1 in M2, M3, and M6 subtypes of AML, the higher levels of LYL1 expression are likely to be associated with the malignant behavior of primary AML cells. This finding is in agreement with the study by Meng et al. on leukemic cell lines, which detected LYL1 expression to increase the growth rate and plating efficiency of the cell lines with differential potential of leukemic cells and showed overexpression of LYL1 in U937 cells to block the all-trans retinoic acid (ATRA)-induced monocytic differentiation. Furthermore, this study demonstrated increased LYL1 expression in erythroid cell populations and suggested that LYL1 might act as a regulator of erythroid differentiation. 

A study by Capron et al. [27] investigated the effect of LYL1 deficiency in mice and showed increased SCL/TAL1 and GATA-1 transcripts in spleen but not in BM-derived erythroblasts with increased hypersensitivity to erythropoietin through increased phenyl hydrazine.

It has been shown that LYL1 overexpression can lead to marked inhibition of the ATRA-induced expression of the monocytic marker CD14 in leukemic cell lines, and that forced expression of LYL1 in K562 cells enhanced spontaneous and hemin-induced erythroid differentiation but blocked spontaneous as well as phorbol myristate acetate-induced megakaryocytic differentiations. Furthermore, increased expression of LYL1 has been reported to enhance resistance to cytarabine in cell lines. 

Our study shows for the first time that there is a significant correlation between the blood counts at diagnosis and overexpression of LYL1. No previous studies gave an idea about this correlation. However, our suggestion may reflect the value of this expression on the degree of maturation of myeloid cells, erythroid cells, and megakaryopoiesis (hematopoietic lineage), and may be explained by the pro-leukemic effect of LYL1 in early hematopoietic progenitors as observed in a study by Lukov et al. Further research on large groups of AML cases is required to clarify this finding. 

Furthermore, we tested whether the LYL1 expression of BM samples at diagnosis could predict the response to induction chemotherapy or the achievement of complete remission. We observed in our study that the rate of complete remission was lower with very high levels of LYL1 expression and that the risk of relapse increased with higher levels of LYL1 expression, suggesting an unfavorable prognosis for cases with enhanced expression. This finding is in line with the results of a study by Meng et al. [28] which revealed that downregulation of LYL1 expression in AML resulted in an increased complete remission rate. Downregulation of endogenous expression of LYL1 in K562 cells by a combination of 3 specific siRNAs could inhibit cellular growth and clonogenicity to some extent, as concluded in the study by Meng et al. It was also postulated in that study that aberrant expression of LYL1 in MDS and AML played a role in the development and phenotype of the diseases by altering the differentiation potential of the cells, increasing the growth rate and plating efficiency of AML cells, and reducing the drug sensitivity. In line with other limited studies, our work points to the value of LYL1 and presents this transcription factor as a new target for therapeutic inhibition in hematological malignancies. These observations could foreshadow a promising tool for a therapeutic target to bHLH protein related to transcription factor as RNA interference targeting specific oncogenes may improve patient outcome in AML, MDS, and CML in blast crisis and will improve the efficacy of chemotherapy and increase the survival rate of those patients. Further work on homogeneous populations with higher numbers of patients and prolonged follow-up is required to clarify the impact of LYL1 overexpression on the prognosis of patients with AML and MDS.

## Figures and Tables

**Table 1:**
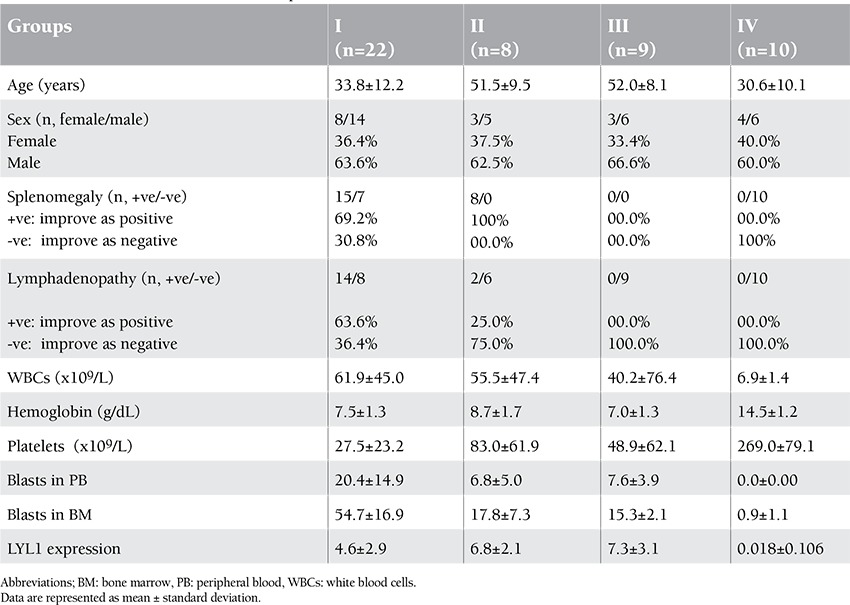
Patient characteristics and LYL1 expression levels

**Table 2:**
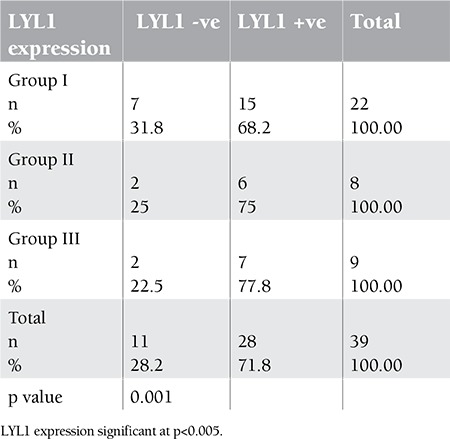
Distribution of LYL1 expression among studied groups

**Table 3:**
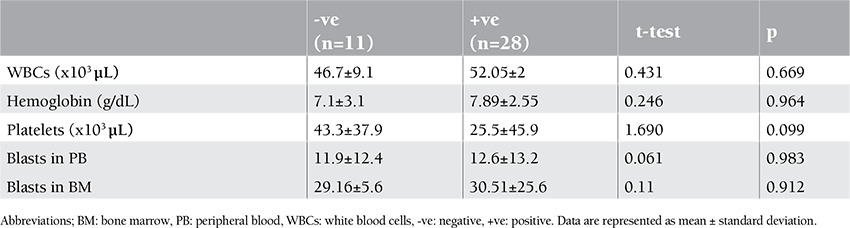
Comparison of hematological data according to positive and negative LYL1 expression

**Table 4:**
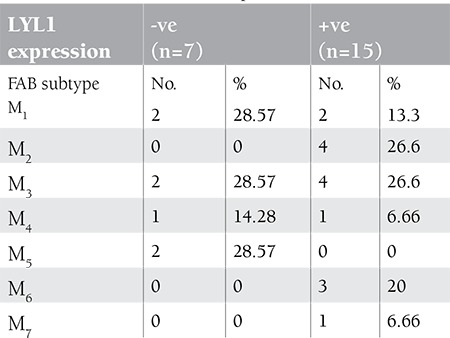
Distribution of patients with AML according to FAB classification and LYL1 expression

**Table 5:**
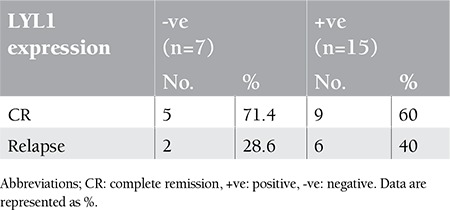
Comparison between % expression of LYL1 and response to induction therapy in AML group

**Table 6:**
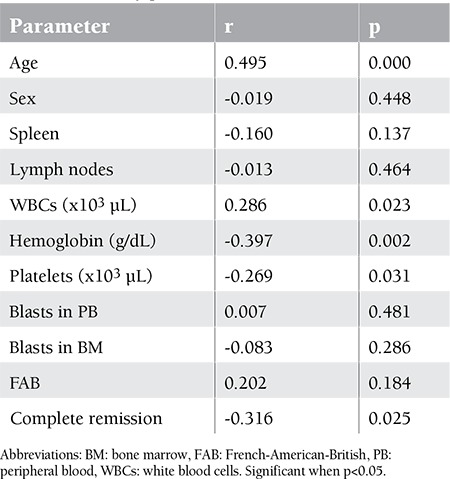
Correlation between LYL1 expression and clinical and laboratory parameters
